# Sebaceous carcinoma in a 54-year-old Black African man after cancer chemotherapy: a case report

**DOI:** 10.1186/s13256-024-04460-z

**Published:** 2024-03-19

**Authors:** Olaejirinde Olaniyi Olaofe, Bolajoko Abidemi Adewara, Chigozie Chidozie Okongwu, Yusuf Olanrewaju Abdullahi

**Affiliations:** 1 Department of Morbid Anatomy and Forensic Medicine, Obafemi Awolowo University, Ile-Ife, Osun State Nigeria; 2 Department of Ophthalmology, Obafemi Awolowo University, Ile-Ife, Osun State Nigeria; 3 Department of Surgery, Obafemi Awolowo University, Ile-Ife, Osun State Nigeria

**Keywords:** Case report, Muir–Torre syndrome, Sebaceous, Carcinoma, Eyelids, African

## Abstract

**Background:**

Sebaceous carcinoma is a very rare malignant skin adnexal tumor that is occasionally aggressive. We have not seen a case of sebaceous carcinoma in our center in the last 10 years. It is extremely rare in Black Africans.

**Case presentation:**

We described the case of a 55-year-old man African man who presented to our ophthalmologist with complaints of growth on the right upper eyelid for 8 months. He had surgery and chemotherapy for rectal carcinoma 6 years prior to presentation and received his last dose of chemotherapy 5 years before seeing our ophthalmologist. There was a history of spontaneous unprovoked bleeding from the lesion. He subsequently underwent surgical excision under general anesthesia. Histology of the mass showed an effaced architecture due to proliferating malignant epithelial cells disposed as trabecules, solid nests, and tongues. The microscopic features of widespread multivacuolated cytoplasm of the neoplastic cells led us to conclude that the tumor was a sebaceous carcinoma. The patient is alive and well.

**Conclusion:**

Sebaceous carcinoma is a rare malignant skin adnexal tumor in Black Africans. It can present as an eyelid mass with spontaneous bleeding. It can follow cancer chemotherapy either because of its association with other tumors in Muir–Torre syndrome or because of mutagenic effects of chemotherapeutic agents.

## Introduction

Sebaceous carcinoma is a rare malignant skin adnexal tumor that is occasionally aggressive [1–3]. It has been reported in various parts of the world and is mainly diagnosed by histological examination of biopsies or resections of the tumor. It has been associated with other malignancies or cancer treatment modalities [4]. We have not seen a case of sebaceous carcinoma in our center in the last 10 years. We present a rare case of sebaceous carcinoma with an episode of spontaneous bleeding in a middle-aged Black African man 5 years after receiving chemotherapy for colorectal cancer.

## Case presentation

A 55-year-old African man presented to our ophthalmologist with complaints of growth on the right upper eyelid for 8 months. Eyelid swelling started from the orbital ridge and progressed to involve the whole upper lid. There was a history of spontaneous unprovoked bleeding from the lesion noticed prior to presentation as well as eye discharge and rashes. He was unable to open the affected eye fully. He had no history of trauma, photophobia, weight loss, or tearing.

He had surgery and chemotherapy for rectal carcinoma 6 years prior to presentation and received his last dose of chemotherapy 5 years before seeing our ophthalmologist. He is a known patient with asthma. There was no family history of similar conditions.

Examination of the right eye revealed a projecting fleshy mass, measuring 3.3 × 2.0 cm, located on the upper eyelid. The mass was firm, lobulated, and nontender with irregular rough surfaces. There was a mildly tender, mobile, nonmatted, right preauricular lymph node.

He subsequently underwent surgical excision under general anesthesia. We confirmed the tumor to be malignant using frozen sections and ensured that the resection margins were free of tumors. The resected lymph node was not involved by the tumor. The surgery was followed by reconstruction of the upper eyelid using the Cutler-Beard procedure. There were no postoperative complications. The patient is alive and well 1 month after the surgery.

**Fig. 1:**
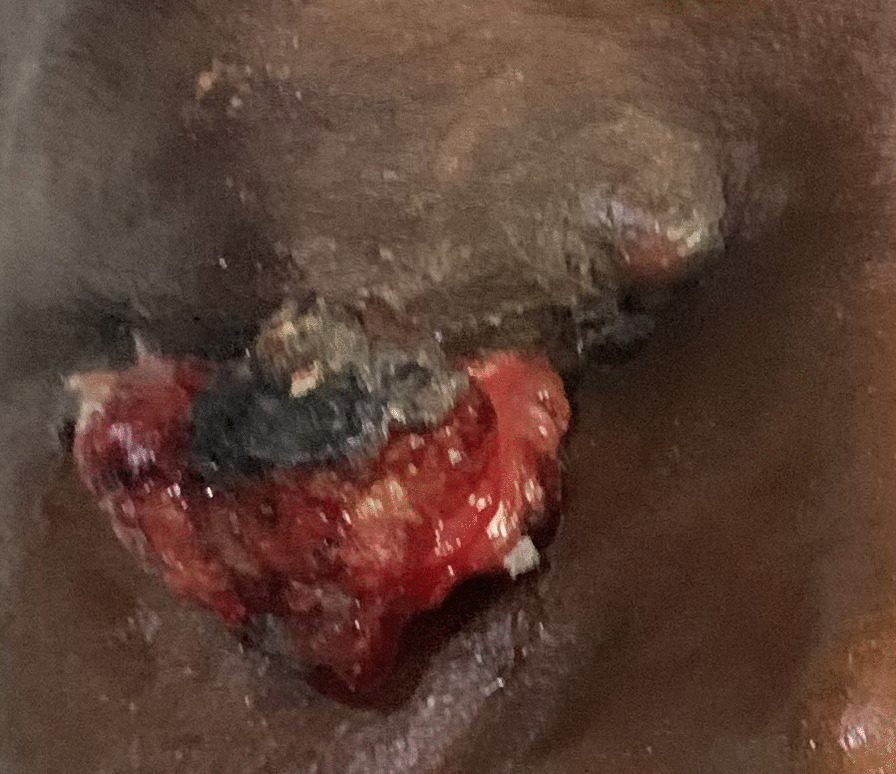
Photograph of the eyes before surgical intervention

**Fig. 2:**
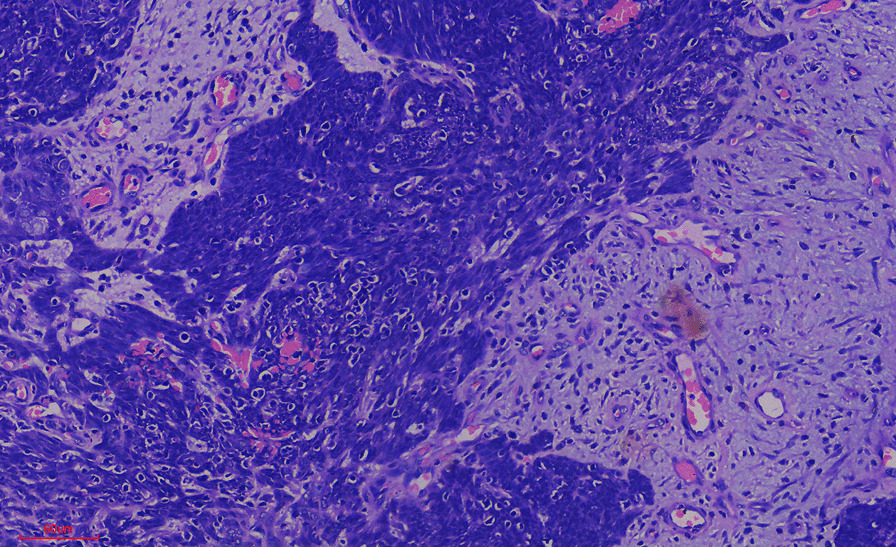
Photomicrograph of stroma infiltration by malignant cells

**Fig. 3:**
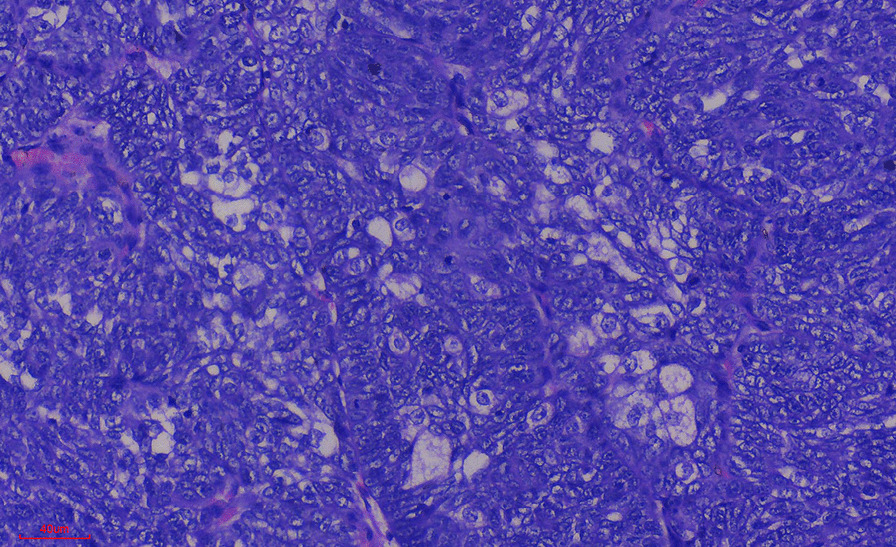
Photomicrograph showing sebaceous differentiation of the malignant cells

Histology of the mass showed an effaced architecture due to proliferating malignant epithelial cells disposed as trabecules, solid nests, and tongues. The individual malignant cells were basaloid, with marked nuclei pleomorphism, irregular nuclei outline, and open vesicular chromatin with prominent nucleoli. Mitoses were up to 11 per high power field with few atypical forms. In some areas, these cells appeared multivacuolated. Areas of squamous differentiation were also present. The features, some of which are shown in Figs. 1, 2, and 3, are conclusive of sebaceous carcinoma. Microscopic examination of the lymph node showed variably sized and shaped lymphoid follicles with few prominent germinal centers. The lymph node was not infiltrated by malignant cells.

Figure 1 shows the right eye with a projecting fleshy mass on the upper eyelid. The mass was firm, lobulated, and nontender with irregular rough surfaces.

Figure 2 shows infiltration of the fibrocollagenous stroma by malignant epithelial cells. There was reactive lymphocytic infiltration and neovascularization. Prolific vascular proliferation could explain the spontaneous bleeding experienced by the patient.

The photomicrograph shows malignant cells with multivacuolated cytoplasm in many areas of the tumor. This is strong evidence of sebaceous differentiation.

## Discussion

The patient’s age is within the age range for sebaceous carcinoma previously reported by some researchers. The tumor is known to occur in the age range of 43–85 years in a study performed on Koreans [5]. Like all cancers, the incidence of this tumor is expected to increase with age, probably owing to the cumulative effects of carcinogenic factors to which the patient is exposed over the years.

Although this malignant tumor is more common in Caucasians, our patient is a Black African. The tumor is very rare locally, as we have not seen any cases in the last 10 years. The rarity of the tumor in our region of the world is consistent with the low incidence of other skin tumors in our region compared with what is reported in the Western world. However, it is difficult to know the true incidence in our area, as many patients do not present to the hospital due to poverty and relatively poor accessibility of high-quality healthcare institutions.

The index case is a male patient who may have had an increased risk of the disease because of his hormonal activity. Although some studies have reported sebaceous carcinoma to be more common in female patients, a few have indicated a preponderance of the disease in male patients [6]. The tumor is known to be androgen receptor positive [7–9]. This can make the tumor incidence higher in male patients. The reduction in estrogen activity after menopause may also contribute to the development of the tumor in postmenopausal women. Men are known to have a more aggressive form of the disease, which may also be related to the increased proliferative effect of androgens. Men with androgen receptor-positive tumors are known to have a poorer prognosis.

Sebaceous carcinoma has been reported in various sites in the body [10–14]. The tumor in the index case was found in the most likely site, which was the ocular region. The mass is known to occur primarily in the ocular region, with less than half occurring in other parts of the body, most especially the head and neck region. It was also situated in the upper lid, which is the most common site for ocular cases. The location of the tumor is probably due to the greater presence of sebaceous glands in the head and neck region of the body, with particularly high concentrations in the eyelids.

Various agents have been postulated as risk factors, including Epstein–Barr virus (EBV) and ultraviolet radiation [13]. The index patient resides in Africa, which has relatively higher sunshine compared with temperate regions. This could have had a contributory role. We are unable to test for EBV to corroborate suggestions by some researchers. In our opinion, it might be necessary to evaluate any possible association of the tumor with the use of eye pencils, as they are commonly applied to the eyelids. However, any possible link is likely to be weak since there is widespread use of eye pencils and very low incidence of the tumor.

Sebaceous carcinoma has been reported following treatment of malignant tumors [15]. The index patient had chemotherapy for colorectal cancer, which could contribute to the genesis of the tumor. Cancer chemotherapeutic agents are well known to be mutagenic and capable of causing secondary malignancies. A case has been reported in a patient on ruxolitinib, a monoclonal antibody previously associated with basal cell carcinoma, Merkel cell carcinoma, and squamous cell carcinoma [15]. Some cases have been associated with Muir–Torre syndrome (MTS), a variant of Lynch syndrome [4]. It is possible that the patient has MTS syndrome. However, we could not carry out genetic tests to confirm MTS as we do not have the facilities.

As is known in typical cases of the tumor, we noticed the presence of lobules and nests that infiltrated the underlying stroma. We observed many basaloid cells. All these findings are consistent with the sebaceous differentiation of the tumor.

The patient had an episode of spontaneous bleeding from the tumor mass. From our background knowledge, this is a rare occurrence. Malignant tumors can have poorly developed leaky vessels that can be easily ruptured. Our case shows that this tumor can cause severe blood loss in patients if not adequately managed.

The individual malignant cells are basaloid (undifferentiated) with marked nuclei pleomorphism, irregular nuclei outline, and open vesicular chromatin with prominent nucleoli. The tumor can easily be identified using morphology when there is extensive sebaceous differentiation, as was seen in this case.

## Conclusion

Sebaceous carcinoma is a rare malignant skin adnexal tumor in Black Africans. It can follow cancer chemotherapy either because of its association with other tumors in Muir–Torre syndrome or because of mutagenic effects of chemotherapeutic agents. It can present as an eyelid mass with spontaneous bleeding. The tumor can easily be identified using morphology when there is extensive sebaceous differentiation, as is seen in this case.

## Data Availability

The tissue blocks are available for future use.
